# PIN2-like proteins may contribute to the regulation of morphogenetic processes during spermatogenesis in *Chara vulgaris*

**DOI:** 10.1007/s00299-016-1979-x

**Published:** 2016-04-11

**Authors:** Aneta Żabka, Justyna Teresa Polit, Konrad Krajewski, Patrycja Paciorek, Jolanta Juszczak, Mateusz Nowak, Janusz Maszewski

**Affiliations:** Department of Cytophysiology, Faculty of Biology and Environmental Protection, University of Łódź, Pomorska 141/143, 90-236 Lodz, Poland

**Keywords:** Callose, *Chara vulgaris*, IAA, PIN2-like proteins, Polar auxin transport, Spermatogenesis

## Abstract

*****Key message***:**

**PIN2-like auxin transporters are expressed, preferentially in a polarized manner, in antheridial cells of freshwater green alga** ***Chara vulgaris*** **, considered to be the closest relative of the present-day land plants.**

**Abstract:**

*Chara vulgaris* represents a group of advanced multicellular green algae that are considered as the closest relatives of the present-day land plants. A highly specialized structure of its male sex organs (antheridia) includes filaments consisting of generative cells, which after a series of synchronous divisions transform into mature sperm, and non-generative cells comprising outer shield cells, cylindrical manubria, and central complex of capitular cells from which antheridial filaments arise. Immunofluorescence observations indicate that PIN2-like proteins (PIN2-LPs), recognized by antibodies against PIN-FORMED2 (PIN2) auxin transporter in *Arabidopsis thaliana*, are expressed in both types of antheridial cells and, in most of them, preferentially accumulate in a polarized manner. The appearance of PIN2-LPs in germ-line cells is strictly confined to the proliferative period of spermatogenesis and their quantities increase steadily till antheridial filaments reach the 16-celled stage. An enhanced level of PIN2-LPs observed in the central cell walls separating two asynchronously developing parts of antheridial filaments (characterized by the plugged plasmodesmata) is correlated with an enhanced deposition of callose. Intense PIN2-LPs immunofluorescence maintained in the capitular cells and its altering polarity in manubria suggest a pivotal role of these cells in the regulation of auxin transport directionality during the whole time of antheridial ontogenesis. Immunohistochemical staining of IAA revealed a clear-cut correspondence between localization sites of auxins and PIN2-LPs. It seems probable then that a supplementary developmental mechanism has evolved in *Chara*, by which all antheridial elements may be integrated at the supra-cellular level via plasma membrane-targeted PIN2-LPs and auxin-mediated processes.

## Introduction

The mechanisms responsible for both biosynthesis and translocation of the phytohormone auxin play a fundamental role in a wide range of processes, such as cell proliferation, elongation and differentiation, through which they exert a considerable impact on the ultimate shape and function of tissues and organs in all vascular plants (Benjamins and Scheres 2008; Lau et al. 2008; Zhao 2010). Although most of the research concerning intercellular polar auxin transport (PAT) and auxin-mediated signaling pathways is still concentrated mainly on the higher plant models (such as *Arabidopsis thaliana*), over the past several years there has also been a growing interest in the evolution of auxin-dependent cellular polarization, correlative interactions, and long-range control of the developmental patterning in early diverged groups of non-vascular algae, liverworts and mosses (Cooke et al. 2002; Fujita et al. 2008; Lau et al. 2009; Boot et al. 2012; Bennett 2015).

The spatio-temporal control over local distribution of indole-3-acetic acid (IAA), the principal and most abundant form of native auxin, is mediated by a group of membrane proteins, including the AUX1/LAX family of influx carriers (reviewed by Swarup and Péret 2012) and auxin efflux carriers, comprising both the PIN-FORMED proteins (PINs) and the ATP-Binding Cassette family B (ABCB) transporters (reviewed by Vanneste and Friml 2009; Křeček et al. 2009; Overvoorde et al. 2010; Zažímalová et al. 2010). In root meristems, intercellular movement and the polarized allocation of IAA are based primarily on the asymmetrical positioning of PIN1 and PIN2 proteins, which are phosphorylated by a PINOID (PID) serine-threonine kinase and dephosphorylated by PP2A phosphatase prior to being targeted to either apical or basal side of the cell (Friml et al. 2004; Michniewicz et al. 2007; Dhonukshe et al. 2010).

Origins of the mechanisms underlying both PAT and auxin-mediated developmental processes have been found in unicellular and multicellular Chlorophyta (Boot et al. 2012) and in some species of the macroscopic brown algae from the phylum Heterokontophyta, a filamentous marine epiphyte *Ectocarpus siliculosus* (Le Bail et al. 2010), having one of the simplest architectures of all multicellular organisms and chosen as a genetic and genomic model of brown algae, *Fucus distichus* (Basu et al. 2002), and *F. vesiculosus* (Polevoi et al. 2003). As judged from an in silico survey, a significant number of genes related to auxin biosynthesis, signaling and transport from land plants have homologs in a relatively small (200 Mb) genome of *E. siliculosus*. Results presented by Le Bail et al. (2010) indicate that IAA-dependent, long-range control over developmental patterning in *E. siliculosus* relies on the same cellular responses as those operating in higher plants, but a signal transduction pathway induced by IAA significantly differs between green plants and the heterokont lineages. Observations in *Fucus distichus* (Basu et al. 2002) and *F. vesiculosus* (Polevoi et al. 2003) clearly indicate an important role of auxin and localized accumulation of IAA in the development of apical basal polarity. The results obtained in both species seem to point that the carrier-mediated auxin efflux contributes to the establishment of temporal and spatial control required for the normal course of morphogenetic events during early stages of embryogenesis in the genus *Fucus*.

Among green algae, Charophytes represent an ancient group of well-organized multicellular ancestors of embryophytes and share numerous morphological and genetic similarities with terrestrial plants (Karol et al. 2001; Piazza et al. 2005; Qiu 2008). When considering morphology, such features comprise rhizoids, a ‘stem-like’ main axis segmented into internodes (covered with files of cortical cells in some members of the Chareae) and nodes, marked by whorls of leaf-like laterals (pleuridia; Pickett-Heaps 1967; Beilby and Casanova 2014). Functional resemblances between characean algae and land plants include three-dimensional apical growth, phragmoplast formation during cytokinesis, chemical composition of cell wall polymers, cell-to-cell communication mediated by plasmodesmata (with actin and myosin-like proteins), and plant hormones (Domozych et al. 2009; Leliaert et al. 2012). Even though relatively little is known about the proteins involved in auxin transport and about the mechanisms of auxin-dependent processes in Charophytes, some data indicate that IAA is synthesized in the apical region of the plant thallus via a tryptophan-independent pathway, and the level of IAA is established by the balance between its rates of production and degradation (Cooke et al. 2002). However, more recent experiments showing 1-*N*-naphthylphthalamic acid (NPA)-sensitive transport of ^3^H-labeled IAA in giant (3–5 cm) internodal cells of *Chara corallina* demonstrate the presence of PAT and, consequently, the occurrence of mechanisms which require the use of specific auxin efflux carriers on the plasma membrane as in higher plants (Boot et al. 2012).

The object of our study is a complex system of generative and non-generative cells which form spherical male sex organs (antheridia) of *Chara vulgaris*. During the early developmental stages, radial multilayer structure of the antheridium is assembled of 8 redundant parts, each composed of only non-generative elements: the outer shield cell, the manubrium, and the group of small capitular cells (Pickett-Heaps 1968). At later stages, an internal organization of the antheridium is established mainly by the circumferential growth of flattened (triangle-shaped) shield cells and the radial growth of rod-shaped manubria towards the central group of primary, secondary and tertiary capitular cells from which germ-line cells develop by repeated budding. At that time, an internal space of the antheridium expands in volume and fills up with antheridial filaments embedded in a glairy mucilaginous fluid released from manubria and capitular cells (Pickett-Heaps 1968; Kwiatkowska and Maszewski 1987a, b). In effect, the external layer of curved, red colored shield cells forms an envelope that protects inner elements of the antheridium and exchange solutes with the external environment (Kwiatkowska et al. 1990). Manubria (protruding from the centers of the shield cells) are thought to produce regulatory factors that induce and control the process of spermiogenesis (Kwiatkowska and Maszewski 1987a; Kwiatkowska 1988). Similar roles have also been ascribed to all types of capitular cells, which operate as transmitters of morphogenetic factors within the whole complex of antheridial cells (Kwiatkowska and Maszewski 1986, 1987b). The non-generative cells acquire their functional identities (associated with a characteristic size, morphology, ultrastructure, and metabolic activity) by means of DNA endoreplication (Kwiatkowska et al. 1990; Maszewski 1991) and developmentally regulated symplasmic cell-to-cell communication (Kwiatkowska and Maszewski 1985; Kwiatkowska 1988; Maszewski and van Bel 1996).

The process of spermatogenesis comprises two ongoing phases. In the first, termed “the proliferative period”, a series of mostly synchronous mitotic divisions multiply the number of haploid spermatids forming antheridial filaments composed of 1, 2, 4, 8, 16, 32, and 64 cells. At each of these developmental stages, characterized by the simplified type of the cell cycle (comprising only S, G2, and M phases), DNA replication remains almost unchanged and begins in late telophase, which implies that the requirements needed for the onset of S phase commence during the preceding interphase. The progressive shortening of successive G2 + M phases, which accounts for most of the variation in cell cycle times (and an increasing contribution of S phase), corresponds with the gradual reduction of cell sizes. Consequently, average loss of cell volume during transition throughout every sequential stage amounts to about 20 %, which means that successive interphase cells reach merely about 4/5 of their volumes attained at previous mitotic division. The second developmental phase, termed spermiogenesis, involves structural reorganization of cells (chromatin condensation and a substantial reduction in the amount of cytoplasm) leading to their terminal differentiation and to the emergence of mature sperm cells (Olszewska and Godlewski 1973; Maszewski and Kołodziejczyk 1991).

A number of previous studies have shown that an integrated approach which aims to explain the mechanisms engaged in spermatogenesis in Characeae is not possible without consideration of structural and functional relations within the whole organized system of cells that form antheridia (Maszewski and Kołodziejczyk 1991; Maszewski and van Bel 1996; Kwiatkowska 2003). From this point of view, the main problem of morphogenesis in male sex organs of *C. vulgaris* must relate to the mode of coordination between the two developmental traits: the first composed of haploid germ-line cells which divide mitotically and, ultimately, undergo terminal differentiation into spermatozoids, and the second, which by increasing the DNA content (via endoreplication) is needed to arrange structural and metabolic properties of relatively large shield cells, manubria, and capitular cells. The spatial character of interactions and the functional links between all component parts of the antheridium suggest that its development may be intimately connected with auxin-mediated mechanisms of morphogenetic patterning. Considering the above and taking into account an inherent relationship between the high proliferative potential of spermatids and the coincident extension of non-generative antheridial cells, the aim of our current study was to investigate the localization of PIN2-LPs as putative mediators of auxin transport during formation of male reproductive organs in *C. vulgaris*.

The observations reported here suggest that proteins with epitopes recognized by antibodies raised against PIN-FORMED2 (PIN2) auxin transporter in *Arabidopsis thaliana* are found in both generative and non-generative cells of male sex organs in *C. vulgaris*. Despite the fact that, as a rule, the intracellular distribution of these proteins corresponds to that reported for the functional PIN2 in the root meristems of higher plants, we find it more convenient to use the term PIN2-like proteins (PIN2-LPs) because of their unknown structure and possible plurality of the binding sites for antibodies used in our studies. The developmentally regulated changes in localization of PIN2-LPs appear well correlated with the coordinated growth of all cellular elements that built an antheridium and perform their IAA-dependent functions both during the proliferative period of spermatogenesis and during terminal differentiation of generative cells into mature spermatozoids.

## Materials and methods

### Plant material

*Chara vulgaris* was collected from monospecific populations in slowly-floating stream in the Arboretum (Rogów Forestry Experimental Station, part of Warsaw University of Life Sciences). In the laboratory, plants were grown in the aquarium at room temperature under natural light (September, 2014). Prior to experimental manipulations, apical parts of thalli with whorls of lateral branches (pleuridia) were washed with sterile distilled water. Seeds of *Arabidopsis thaliana* (Col-0; obtained from the Laboratory of Plant Molecular Biology, Institute of Biochemistry and Biophysics, Polish Academy of Sciences, Warsaw, Poland) were surface-sterilized with 70 % (v/v) ethanol for 3 min and 10 % (v/v) bleach with 0.01 % (v/v) Triton X-100 for 5 min.

### Immunoprecipitation of PIN2 (*A. thaliana*) and PIN2-LPs (*C. vulgaris*) and protein blotting

Immunoprecipitation and protein blot assays for PIN2-LPs extracted from apical parts of *C. vulgaris* carrying whorls with young antheridia and (as a control) for PIN2 proteins extracted from root tips of *A. thaliana* (0.5–1 mm in length) were performed according to methods described earlier (Żabka et al. 2015). Briefly, excised plant materials were lysed using a P-PER Plant Protein Extraction Kit (Pierce, Rockford, IL, USA) containing Protease Inhibitor Cocktail (P-9599; Sigma-Aldrich) and the extracts were cleared afterwards by centrifugation. For immunoprecipitation (carried out according to the supplied protocol), Dynabeads^®^ Protein A (Novex, Life Technologies) was incubated with diluted chicken polyclonal anti-PIN2 primary antibody (Agrisera) and the obtained complexes were suspended in crude cell lysates. Dynabeads^®^-antibody-antigen aggregates (washed with Washing Buffer) were suspended in Elution Buffer for 10 min at 70 °C. Protein samples were fractionated on 4–12 % Bis–Tris 2-(4-morpholino)-ethanesulfonic acid SDS–NuPAGE Novex gel (Invitrogen Corp., Carlsbad, CA, USA), blotted onto polyvinylidene fluoride membrane (0.2-mm pore size; Invitrogen) and detected using the same anti-PIN2 primary antibody (diluted 1:2000) and the Chromogenic protein blot Immuno-detection Kit (Invitrogen).

### Immunolocalization of PIN2-LPs in antheridial cells of *C. vulgaris*

Immunofluorescence detection of PIN2-LPs in generative and non-generative antheridial cells of *C. vulgaris* using antibodies raised against synthetic peptides corresponding to AtPIN2 was carried out according to the method described by Rahman et al. (2010) with some modifications. Apical parts of *C. vulgaris* thalli were fixed for 45 min in 50 mM PIPES-buffered (pH 7.0) 4 % paraformaldehyde solution (with the addition of 0.5 mM CaCl_2_) and permeabilized in MTSB (50 mM PIPES, 5 mM EGTA, 5 mM MgSO_4_, pH 7.0; Sigma) containing glycerol (10 %) and Triton X-100 (0.2 %). After brief treatment with cold methanol (−20 °C) and rehydration in MTSB, pleuridia carrying antheridia at various developmental stages were macerated according to Bannigan et al. (2006) for 15 min with citrate-buffered mixture (pH 5.0; 38 °C) containing 0.1 % pectinase from *Aspergillus niger* (Fluka) and 0.01 % pectoliase Y-23 (ICN). After that, isolated antheridia were incubated with 10 % (v/v) DMSO and 3 % (v/v) Nonidet P-40 in MTSB for 1 h, rinsed with MTSB (3 × 5 min) and treated for 1 h with 3 % BSA and 0.01 % sodium azide (blocking solution). Then they were squashed onto Super Frost Plus glass slides (Menzel-Gläser, Germany) to release rosettes of antheridial filaments adjoined to non-generative cells and air dried. Slides were incubated overnight at 4 °C with the chicken polyclonal anti-PIN2 primary antibody dissolved with 3 % BSA in MTSB (1:1000, Agrisera), washed with the same buffer and incubated for 3 h with the secondary rabbit anti-chicken FITC-conjugated antibody (1:600, Sigma). Omitting the primary antibody on some of the slides served as a negative control.

### Immunodetection of IAA

Apical parts of *C. vulgaris* thalli were immediately fixed in a fresh 3 % aqueous 1-ethyl-3-(dimethyl-aminopropyl)-carbodiimide hydrochloride (EDAC, Sigma) for 1 h (4 °C) and, after washing with cold PBS, postfixed for 3 h in a 0.5 % (v/v) glutaraldehyde and 3 % (w/v) paraformaldehyde mixture in PBS (4 °C). Following maceration (performed using pectinase/pectolyase mixture, as described earlier), isolated antheridia were squashed onto glass slides and treated with PBS-based blocking buffer, containing 0.1 % Tween-20 (v/v), 1.5 % glycine (w/v) and 5 % BSA (w/v). The slides, processed according to Chiappetta et al. (2009), were incubated overnight with diluted (1:300) mouse anti-IAA monoclonal primary antibody (Sigma) in PBS/BSA solution (0.8 %, w/v; 4 °C), washed and incubated again for 3 h with 1:100 (the same buffer) goat anti-mouse alkaline phosphatase conjugated secondary antibody (Sigma). After washing, slides were treated with NBT (nitro blue tetrazolium) and BCIP (5-bromo-4-chloro-3-indolylphosphate) mixture for 5 min, then rinsed with stop buffer (100 mM Tris–HCl, pH 8.0; 1 mM EDTA) and embedded in PBS/glycerol mixture.

### ER labeling with DiOC6(3) and callose staining with aniline blue

ER labeling with 3,3′-Dihexyloxacarbocyanine Iodide (DiOC6(3); Life Technologies), and callose staining with aniline blue (Sigma) solution were performed on live antheridia of *C. vulgaris*. DiOC6(3) was dissolved in ethanol to 1 % (w/v) and then in water to a final concentration of 5 μg/mL. Callose was stained with sodium phosphate buffered (0.07 M, pH 9) aniline blue (AB) solution. Whole pleuridia were cut off the thalli and stained at room temperature for 7 min with DiOC6(3), or for 5 min with AB. After that, antheridia at different stages of spermatogenesis were isolated, briefly rinsed with water, gently spread onto glass slides under the coverslips and observed immediately under the microscope.

### Observations and analyses

Observations were made under an Eclipse E-600 epifluorescence microscope (Nikon, Japan) using B2 filter (blue light; *λ* = 465–496 nm) for the rabbit anti-chicken FITC-conjugated antibody and DiOC6(3), and U2 filter (UVB light; *λ* = 340–380 nm) for DAPI- and AB-stained cells. All images were recorded at exactly the same time of integration using DS-Fi1 CCD camera (Nikon, Japan). Axial scans taken along representative cell files were plotted using ImageJ software. Confocal observations were made with Leica SP8 (Germany) using laser lines 405 nm (DAPI) and 553 nm (Alexa Fluor^®^ 555). Images were acquired with HCPL APO 63×/1.40 oil. Confocal microscopy was performed in the Laboratory of Microscopic Imaging and Specialized Biological Techniques at the Faculty of Biology and Environmental Protection, University of Lodz. The cell cycle positions (if needed) were estimated by morphometric analysis (cell length measurements) combined with the cytometric analysis of nuclear DNA (*C* value) after DAPI staining. All immunofluorescence analyses and Western blot assays were repeated at least twice, while other experiments were repeated several times. For statistical analysis, the intensities of immunofluorescence were measured for 50 cell wall areas in 50 (2 cell stage) and 25 (later stages) individual filaments. One-way ANOVA and Bonferroni post hoc test were applied.

## Results

### Immunodetection of PIN2-LPs in antheridial cells of *C. vulgaris*

**Fig. 1:**
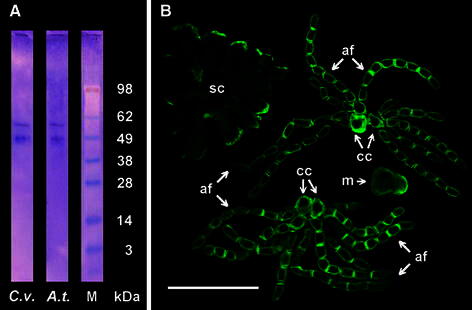
Immunodetection of PIN2 proteins. **a** Protein blots following immunoprecipitation of PIN2 (PIN2-LPs) from apical parts of *Chara vulgaris* thalli (*C.v.*) and from root tips of *Arabidopsis thaliana* (*A.t.*); *M* molecular weight marker, *kDa* mass scale. **b** Confocal microscopy images of *C. vulgaris* antheridial cells following immunolocalization of PIN2-LPs; *af* antheridial filaments, *sc* shield cell, *cc* capitular cells, *m* manubrium. *Scale bar* 75 μm

Prior to our study on generative and non-generative antheridial cell systems, the specificity of polyclonal antibody raised against synthetic peptides derived from AtPIN2 sequences (encoded by the locus At5g57090) was tested by immunoprecipitation (IP) using total protein extracts from both apical parts of *Chara* thalli and root tips excised from *Arabidopsisthaliana* seedlings (as previously described; Żabka et al. 2015). For each of these samples, IP followed by Western blot assay revealed two bands at about 49 and about 60 kDa (Fig. 1a), corresponding to molecular weight of AtPIN2 determined by Müller et al. (1998). Furthermore, in all types of antheridial elements (with the exception of nearly ball-shaped capitulate and triangle-shaped shield cells), an increased accumulation of PIN2-LPs immunofluorescence was preferentially localized to the plasma membrane region of the transverse cell walls (Fig. 1b), and as will be shown later, often associated with its asymmetric distribution, which is characteristic of the auxin carrier proteins.

### Distribution patterns of PIN2-LPs in antheridial filaments

**Fig. 2:**
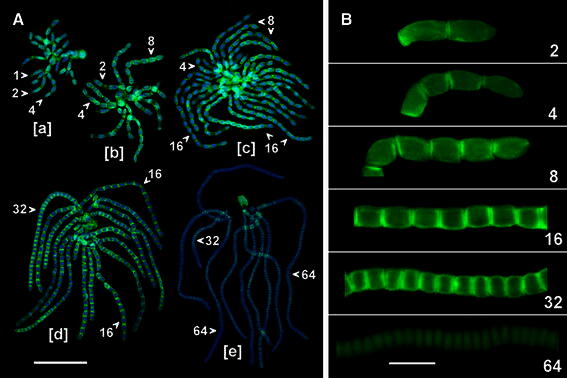
Immunfluorescence of PIN2-LPs during development of antheridial filaments in *C. vulgaris*. Selected filaments are indicated (*arrowheads*) by the number of component cells (spermatids). **a** Rosettes of filaments at early [*a*], middle [*b*, *c*], and late [*d*] stages of the proliferative period and at the onset of differentiation into mature sperm cells [*e*]. Nuclear DNA stained blue with DAPI. *Scale bar* 75 μm. **b** Antheridial filaments at 2- to 64-cell stages (numbers) showing differences in the intensity of PIN2-LPs immunofluorescence localized to the transverse cell wall regions between adjacent spermatids. *Scale bar* 20 μm

During preparation for microscopic examination, antheridial filaments squashed onto slides form a rosette-like complexes associated with their progenitor (capitular) cells (Figs. 1b, 2a). Each of such sets, usually representing 3 (or rarely more) successive developmental stages (generations) of spermatids, comprises 1–2–4-celled filaments (early proliferative period; Fig. 2a[a]), 2–4–8- and 4–8–16-celled filaments (middle proliferative period; Fig. 2a[b, c]), and 16-, 32-celled filaments (late proliferative period; Fig. 2a[d]). Most commonly, at the onset of spermiogenesis, rosettes of antheridial filaments are composed of 32, and 64 cells (Fig. 2a[e]) that gradually develop into mature spermatozoids. As evidenced, PIN2-LPs are localized to the transverse cell wall regions of antheridial filaments throughout all stages of the proliferative multiplication of spermatids (Fig. 2a[a–d]), which is in marked contrast to their final, ‘mitotically silent’ stages of terminal differentiation (Fig. 2a[e]).

**Fig. 3:**
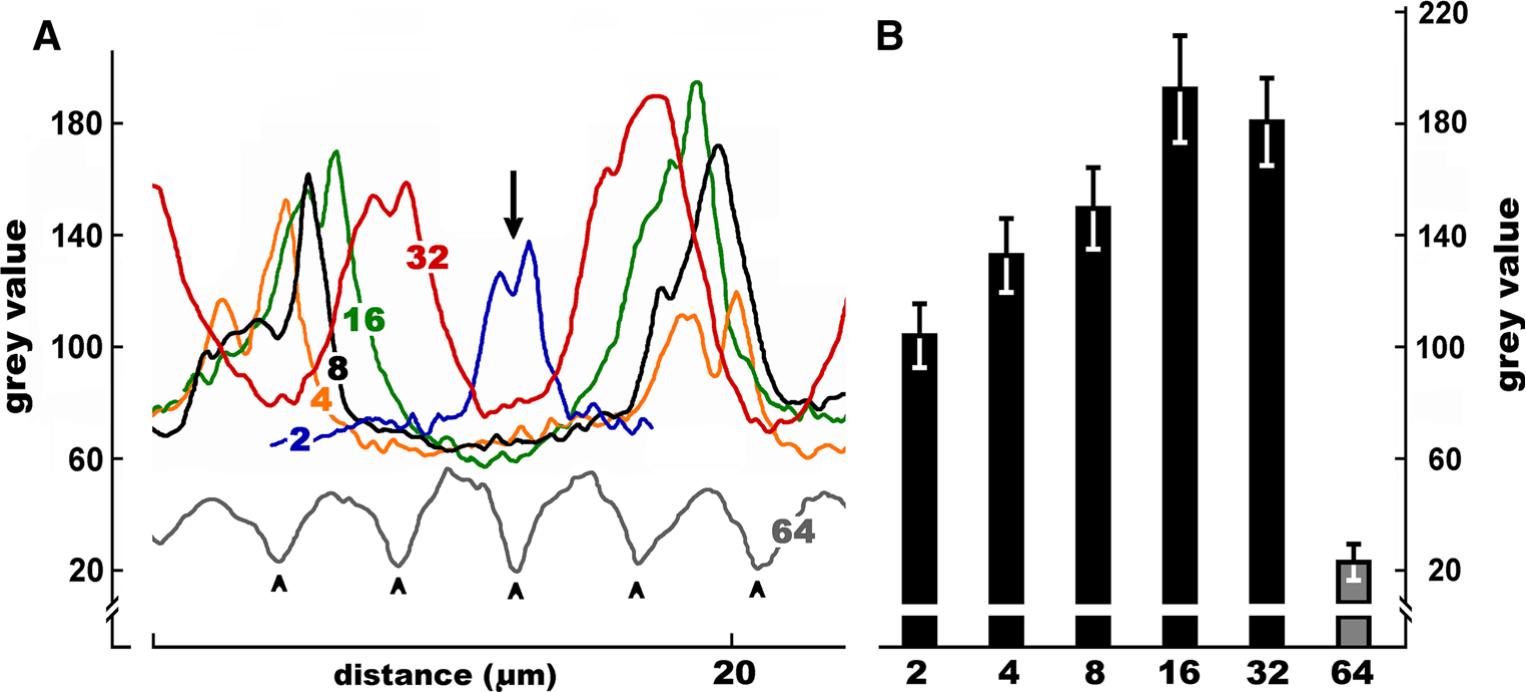
Changes in the intensity of PIN2-LPs immunofluorescence (expressed as *grey* value) evaluated for successive developmental stages of antheridial filaments. **a** Representative single axial scans across transverse cell wall areas in selected 2-, 4-, 8-, 16-, 32-, and 64-celled antheridial filaments (developmental stage indicated by the inserted number). For the two-celled antheridial filament a single cell wall region was scanned (*middle plot*; *arrow*), for each of the 4–32 cell stages two cell wall regions were scanned, and for the 64-celled stage (early spermiogenesis) five cell wall regions were scanned (*arrowheads*). **b** Mean intensity (±SD) of PIN2-LPs immunofluorescence evaluated for cell wall regions in antheridial filaments during successive stages of the proliferative period (2–32 cell stages; *black bars* in the diagram) and during early spermiogenesis (64 cell stage; grey bar in the diagram). The data were statistically evaluated using one-way ANOVA (*F*_(5,354)_ = 1130.54, *p* < 0.001) and then with Bonferroni post hoc test. The Bonferroni test has shown statistical significance for all analyzed data sets at the *p* < 0.001 level

**Fig. 4:**
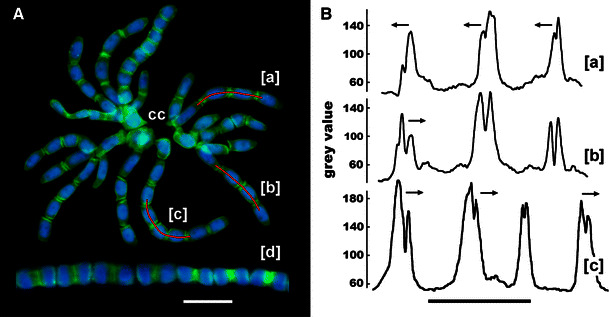
Polar localization of PIN2-LPs at cell wall regions in antheridial filaments. **a** The rosette composed of one- to eight-celled antheridial filaments at the early/middle stage of the proliferative period of spermatogenesis, attached to a complex of capitular cells (cc); nuclear DNA stained blue with DAPI. [*a*–*c*] Immunofluorescence of PIN2-LPs axially scanned along the lines drawn in selected four- [*a*, *b*] and eight-celled filaments [*c*], shown as plots in (**b**); [*d*] 32-celled antheridial filament presenting PIN2-LPs in phragmoplasts formed during late telophase. *Scale bar* 20 μm. **b** Axial scans demonstrating various modes of PIN2-LPs deposition on either sides of transverse cell walls separating successive spermatids: [*a*] asymmetrical, oriented towards the capitular cell, [*b*] mixed, oriented towards the apical cell of the filament (on the left cell wall area) and then followed by two cell walls showing (on both sides) equal quantities of PIN2-LPs, and [*c*] a dominant centrifugal distribution of PIN2-LPs (oriented towards the apical cell of the filament in all but one scanned cell wall areas). *Arrows* indicate a hypothetical direction of the net auxin flow. *Scale bar* 20 μm

Although no statistically significant variations in the intensities of PIN2-LPs labeling were found between different phases of the cell cycle (S-, G2-, or M phase), a considerable increase in the immunofluorescence signal was observed in antheridial filaments while their passing through successive stages of development (Fig. 2b). This effect is reflected in the microfluorimetric axial scans taken across representative cell wall regions (Fig. 3a) and in the mean values calculated for the interface areas connecting the neighboring cells in consecutive generations of spermatids (Fig. 3b; note an almost 80 % rise in immunofluorescence of PIN2-LPs during the transition from the 2- to the 16-cell stage). Direct microscopic observations and microfluorimetric scanning of antheridial filament cells (irrespective of the developmental time at which they were analyzed) revealed an asymmetric distribution of these proteins, with nearly 46 % of all filaments having their PIN2-LPs rich cell wall regions oriented basipetally (facing towards the capitular cell), about 25 % of filaments showing an opposite polarization (towards an apical cell of the filament), and slightly more than 29 % of filaments demonstrating roughly equal (bipolar) allocation of PIN2-LPs (Fig. 4a, b). Additionally, an enhanced affinity of these proteins for the internuclear region was observed during telophase (mostly at later developmental stages; Fig. 4a[d]), suggesting some kind of functional relationship between PIN2-LPs and the central area of the cell occupied by the phragmoplast.

**Fig. 5:**
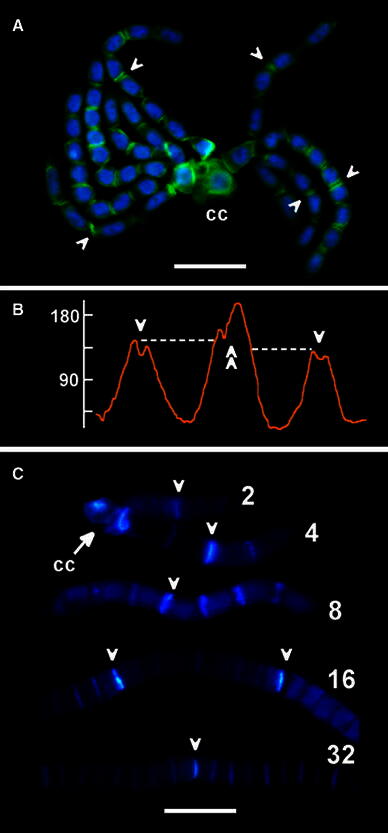
Increased concentration of PIN2-LPs in antheridial filaments correlates with an enhanced callose deposition at the early formed cell walls. **a** The rosette of two-, four-, and eight-celled antheridial filaments adjoined to the central complex of capitular cells (cc); the areas of cell walls expanded during the 1–2 cell stage transition (created in the past during the early proliferative period and now being the oldest) reveal increased amounts of PIN2-LPs (*arrowheads*). *Scale bar* 25 μm. **b** Axial scan after PIN2-LPs immunofluorescent labeling (*Y* axis, *gray* values) taken at a distance of 25 μm in the central region of the 16-celled antheridial filament; note the difference between the intensities of signals obtained at the middle cell wall (*double arrowhead*) and at the two neighboring cell wall areas (*single arrowheads*). **c** Aniline blue staining of callose localized to transverse cell walls in antheridial filaments at various developmental stages (indicated by the inserted cell numbers). Early-formed cell walls pointed out by *arrowheads*. *Scale bar* 20 μm

The intrafilamentous distribution of PIN2-LPs immunofluorescence signals is further complicated by an asynchronous development of antheridial filaments due to transient blockage of plasmodesmata which penetrate transverse cell walls to establish cytoplasmic continuity between individual spermatids (Kwiatkowska and Maszewski 1985, 1986; Lucas et al. 1993). According to the early findings in *Chara contraria* (Teleżyński 1929), the most common kind of such an asynchrony is observed in antheridial filaments composed of only two groups of cells, each of them preserving their own (inner) synchronization of cell cycle-regulated events. As evidenced later by electron microscopy in *Chara vulgaris* (Kwiatkowska and Maszewski 1976, 1985, 1986), the two types of transverse cell walls distinguished in these filaments include those linking synchronous cells into a symplastic continuum of spermatids (equipped with open plasmodesmata) and those characterized by an increased thickness, which separate two or more asynchronous parts of the same filament (equipped with plasmodesmata plugged by an electron-dense material). Immunofluorescence microscopy combined with the axial scan measurements revealed that nearly all cytoplasmic regions adjacent to the latter type of cell walls (the oldest ones, formed at the earliest developmental stages) are characterized by a relatively high amount of PIN2-LPs (Fig. 5a, b). As determined by vital staining of antheridial filaments with aniline blue and fluorescence microscopy (Fig. 5c), these same walls display an excessive accumulation of callose, functioning probably as a supplementary mechanism for the control of symplastic cell-to-cell communication at the border between two asynchronous parts of the filament.

**Fig. 6:**
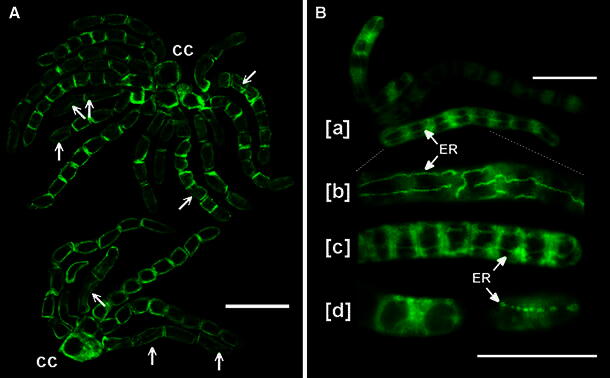
Immunofluorescence labeling of PIN2-LPs reveals tubular structures which may correspond to DiOC6(3)-stained cisterns of endoplasmic reticulum. **a** Confocal images of PIN2-LPs in two rosettes of two-, four-, and eight-celled antheridial filaments attached to a complex of capitular cells (cc); spermatids displaying tubular structures are indicated (*arrows*). *Scale bar* 20 μm. **b** Live antheridial filaments stained fluorescently with DiOC6(3) under the conventional fluorescence microscope. [*a*] Portion of a rosette of filaments with cisterns of endoplasmic reticulum (ER). *Scale bar* 25 μm. [*b*] Enlarged fragment of the eight-celled antheridial filament shown in [*a*]. [*c*] Portion of antheridial filament at late stage of the proliferative period showing DiOC6(3)-stained ER cisterns. [*d*] Apical cells of an antheridial filament at different focal planes displaying nuclear envelope membranes (*left*) and a row of vesicular ER compartments (*right*). *Scale bar* for [*b*–*d*] 25 μm

While the prolonged maceration of *C. vulgaris* resulted in a relatively poor morphology of most generative and non-generative cells, it also appeared beneficial by providing better immunofluorescent visualization of thin and elongated structures, recognized mainly in rosettes of antheridial filaments at relatively early stages of spermatogenesis. As shown in Fig. 6a (arrows), some of them extend between or span two opposite (transverse) cell walls. Mutual contact of these structures, originating from neighboring cells (seen in certain cases only), suggests that they probably represent cisterns of endoplasmic reticulum (ER), which at times may even traverse across the plasmodesmatal channel as a ‘desmotubule’ (or the ‘central rod’) linking adjacent areas of the cytoplasm (Demchenko et al. 2014). Certain support for this assumption comes from fluorescence images of live antheridial filaments stained with DiOC6(3), a lipophilic cationic dye known to localize predominantly to the intracellular membrane systems comprising ER, ER-derived vesicles and mitochondria (Quader et al. 1989; Terasaki 1989). The micrographs in Fig. 6b illustrate large ER cisterns extending over the length of many spermatids, which may suggest their role in establishing a tubular system that interconnect distinct cells functioning synchronously either as part of or as the whole filament.

### Distribution patterns of PIN2-LPs in non-generative antheridial cells

**Fig. 7:**
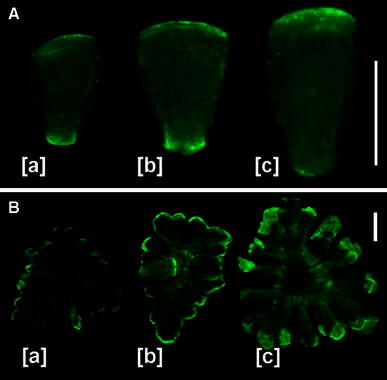
Immunofluorescence of PIN2-LPs in manubria and shield cells. **a** Localization of PIN2-LPs in manubria depends on the developmental period of an antheridium. During the early stages of the proliferative period [*a*], PIN2-LPs are confined mostly to the shorter cell wall connected with the central complex of capitular cells, while at later stages [*b*, *c*] the dominant polarization of PIN2-LPs changes towards the longer transverse cell wall adjoined to the shield cell. *Scale bar* 40 μm. **b** The quantity of PIN2-LPs localized to the multi-lobed outer cell walls of the shield cell gradually increases during successive phases of antheridial development [*a*, *b*], reaching maximum at late stages of spermatogenesis [*c*]. In [*b*], manubrium attached to the central part of the shield cell is visible. *Scale bar* 40 μm

Specific PIN2-LPs immunofluorescence was observed in every type of non-generative antheridial cells and, with the exception of manubria, its intensity and localization showed only minor variation throughout the whole period of spermatogenesis. All generations of capitulae revealed an intense diffuse staining pattern over the entire cell with some increase in labeling at those portions of cell walls which are adjoining to the antheridial filament cells (Figs. 1b, 2a[a–e], 4A, 5A, 6A). The most dynamic changes in both the intensity and spatial distribution of PIN2-LPs immunofluorescence were seen in manubria (Fig. 7a). At the early developmental stages of spermatids (up to the four-cell stage), nearly all manubria (89 %) displayed the polarized accumulation of PIN2-LPs in the proximal cell wall regions close to the primary capitular cells (Fig. 7a[a]). In antheridia containing 8- and 16-celled filaments, a significant number of manubria showed bipolar distribution of PIN2-LPs (41 %; Fig. 7a[b]), while the rest of them revealed PIN2-LPs concentrated (at roughly equal proportion) to either one or the other pole of the cell. At later stages (i.e., by the end of the proliferative period and during spermiogenesis), a unipolar localization of these proteins appeared again, this time, however, directed towards the distant cell wall region facing the center of the shield cell (Fig. 7a[c]). During all stages of spermatogenesis, shield cells displayed an increasing intensity of PIN2-LPs immunofluorescence localized to the peripheral areas of multi-lobed edges and (to a lesser extent) to the central, perinuclear regions (Fig. 7b[a–c]).

### Distribution patterns of IAA in antheridial filaments and non-generative antheridial cells

**Fig. 8:**
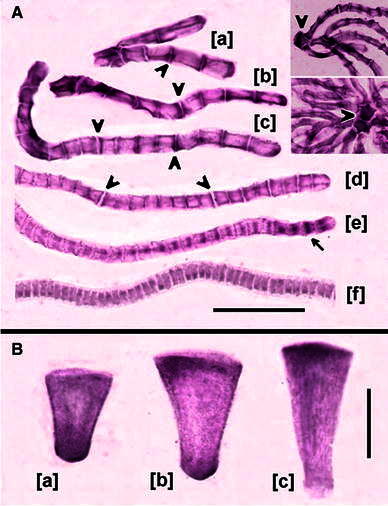
Immunohistochemical staining of antheridial cells of *C. vulgaris* using anti-IAA and AP-conjugated antibodies. **a** Antheridial filaments during successive developmental stages: [*a*] 2- and 4-celled filaments, [*b*, *c*] 8- and 16-celled filaments, respectively, [*d*, *e*] 32-celled filaments, [*f*] 64-celled filament; [*e*] an asynchronous filament composed of telophase cells showing immunolocalization of IAA to the phragmoplasts.* Insertions* presented at lower magnification in the upper right corner show antheridial filaments adjoined to the heavily stained capitular cells (*arrowheads*). *Scale bar* 30 μm for [*a*–*f*] and 50 μm for the* insertions*. **b** Localization of IAA in manubria varies depending on the developmental period of an antheridium. During the early stages of the proliferative period [*a*], IAA is asymmetrically distributed, close to the shorter wall of the manubrium connected with the complex of capitular cells; at later stages [*b*, *c*] polarization of IAA changes towards the longer transverse cell wall of the manubrium, which is adjoined to the shield cell. *Scale bar* 30 μm

Despite the relatively poor preservation of antheridial cells in samples of *C. vulgaris* thalli fixed for immunohistochemical detection of auxin, the overall distribution pattern of IAA identified by immunolocalization with anti-IAA (AP; Fig. 8) has been found to correspond well with the localization of PIN2-LPs. Accordingly, in the generative cells (spermatids), the most intense staining was associated with the transverse walls, especially those separating asynchronous halves (Fig. 8a[a, b]) or quarters of the antheridial filament (Fig. 8a[c, d]). As in the case of PIN2-LPs, IAA labeling was observed throughout early and middle stages of the proliferative period of spermatogenesis, with a slightly fainter staining in the 32-celled filaments (Fig. 8a[d]); no labeling was apparent during the succeeding stages of cells’ differentiation into spermatozoids (Fig. 8a[f]). In addition, similarly to PIN2-LPs (mostly at late proliferative stages), AP staining was seen in the internuclear areas occupied by the expanding phragmoplasts in the telophase cells (Fig. 8a[e]). In contrast to immunofluorescence of PIN2-LPs, however, high amounts of IAA were found already in the two- and four-celled filaments (Fig. 8a[a]), and the polarization of IAA at either the apical or basal side of the cell was generally less evident.

A correspondence between the immunohistochemical staining of IAA and immunofluorescent labeling of PIN2-LPs could be clearly recognized in only two types of non-generative antheridial elements, i.e., capitulae and manubria, since the distortion of shield cells did not allow for adequate comparisons. Consequently, relatively large quantities of blue AP precipitates have been localized in almost all capitular cells (of the 1st and the 2nd order; Fig. 8a, inserted frames), suggesting that their high concentrations of auxin and PIN2-LPs are causally linked. Such connections become even more convincing in manubria, showing differences in the distribution (basal, bipolar, or apical) of the majority of detectable IAA (Fig. 8b; compare with Fig. 7a).

## Discussion

As in many other complex and dynamic plant cell systems, male sex organs in *C. vulgaris* exhibit coherent behavior: their generative and non-generative parts are highly coordinated and operate in an extraordinarily integrated fashion. This coherence is achieved primarily by two principal mechanisms: different dynamics of DNA endoreplication, which constitutes an effective strategy for cellular growth (Maszewski 1991), and the extensive changes in plasmodesmata structure, which are responsible for the altering connectivity between antheridial cells (Kwiatkowska 1999). The results presented in our current study indicate that the occurrence and distribution of PIN2-LPs may contribute to the developmental program of spermatogenesis by additional regulatory capacities based on the polarized transport of auxin.

Prior to discussing specific problems relating to the role of IAA and PAT in the morphogenetic development of male sex organs in *Chara*, at least two important issues need to be considered in a wider evolutionary context. First, our results seem to indicate that PIN proteins localized in *C. vulgaris* belong to the subfamily of canonical ‘long’ PINs containing multiple regions of highly conserved residues (Zažímalová et al. 2010; Bennett 2015). This would be consistent with the implication for a sister-group relationship between the Charales and the land plants (e.g. based on the whole mitochondrial genome analysis of *C. vulgaris*; Turmel et al. 2003), and with the arguments that similarities in their complex development are shared ancestral characteristics. It is conceivable then that the same may be true for PAT and, accordingly, for PIN proteins in Charales, which perhaps are even more compatible with canonical PINs than with PIN proteins from other members of Charophyta, such as *Klebsormidium flaccidum* and *Spirogyra pratensis* (Bennett 2015).

Immunolocalization of PIN2-LPs in *C. vulgaris* raises another problem that cannot be conclusively solved until more and better evidence is available. Strong linear deposition of these proteins in close contact with the transverse cell walls of antheridial filaments (also seen in manubria) suggests that PIN2-LPs are predominantly membrane-associated, even though their presence in ER is likely as well, as indicated by our immunofluorescence study. This finding seems noteworthy considering recent data showing that, in the model moss *Physcomitrella patens*, an effective auxin transport mediated by PIN transporters is diversified into ER- and plasma membrane-localized PIN proteins (Viaene et al. 2014). Whereas ER-localized PINs are thought to maintain auxin homeostasis within cells, most of *P. patens* PINs are plasma membrane targeted and may reveal polarized localization (Bennett et al. 2014; Bennett 2015). The other question refers to the bipolar localization of immunofluorescence signals observed in almost all spermatids, irrespective of developmental stage and position in the cell cycle. Most often, PIN2-LPs are more abundant on one and the same side of each cell than on the other. The redundancy of such an arrangement may contribute to the formation of a coordination mechanism, by which long-distance directional flux of auxin can be established over the whole filament or its synchronous fragment. However, bipolar distribution of PIN2-LPs is in contrast to the asymmetric allocation of PIN2 efflux carriers in root meristems, i.e. accumulated either exclusively to the apical membrane of epidermal cells, or exclusively to the basal membrane of cortical cells (Feraru and Friml 2008; Laskowski et al. 2008). The localization pattern of PIN2-LPs in *C. vulgaris* may thus be regarded either as “specific” (adapted to the structural and functional features of the antheridium), or “primitive” (precursory to later, more defined organization of auxin transport in higher land plants).

When considering the developmental aspects of the germ-line cells in the time context, a strong contrast is apparent between the proliferative period, characterized by an increasing expression of PIN2-LPs, and spermiogenesis, during which these proteins were undetectable by immunofluorescence microscopy. It seems reasonable to assume that this kind of difference is directly linked to an inherent biological program, initiated in an auxin transport-dependent manner, and then accomplished via PAT-unrelated differentiation towards functional sperm cells. Such a view is consistent with the earlier observations of the auxin-mediated signaling pathways involved in cell growth and mitotic divisions in antheridial filaments of *C. vulgaris*. As evidenced by Godlewski (1980), exogenously applied IAA (in concentrations ranging from 10^−7^ to 10^−5^ M) induces a pronounced shortening of the proliferative period, mostly due to the accelerated G2 and M phase transitions. These same experiments indicated that IAA increases, while PCIB (an inhibitor of auxin action) decreases mitotic activity already after 2–4 h treatments. Interestingly, in IAA-treated plants, a relatively more significant reduction of both phases was noted in the 16- and 32-celled filaments, as compared with the earlier stages represented by shorter filaments consisting of two-, four, and eight-cells. It can be assumed, then, that a gradual increase in PIN2-LPs level observed in our study, may play a direct role in establishing the required dynamics of auxin transport, and consequently, may have significant impact on the time-course modifications of successive cell cycles before the onset of spermiogenesis.

An enhanced accumulation of PIN2-LPs in the vicinity of transverse cell walls that separate asynchronous parts of antheridial filaments is still another problem to be mentioned here. In addition to the impaired capacities for symplasmic cell-to-cell delivery of signaling agents (due to electron-dense homogeneous substance inside plasmodesmata; Kwiatkowska and Maszewski 1976, 1986), the specific interface walls adjoining semi-autonomous groups of spermatids reveal large amounts of callose (β-1,3-glucan), as defined by fluorescence after aniline blue staining. This observation deserves special interest in view of the emerging interrelationships between cell components that are engaged in polar auxin transport, callose metabolism and plasmodesmatal functions. As regards this aspect, earlier research has shown that two enzymes, i.e. GLUCAN SYNTHASE LIKE 8 (GSL8; involved in callose synthesis) and β-1,3-glucanase (involved in callose turnover), both play an essential role in the callose-mediated regulation of plasmodesmatal connectivity between the cells (Levy et al. 2007; Chen and Kim 2009). The experiments performed by Han et al. (2014) indicate that there is an auxin-GSL8 feedback loop which restricts symplasmic diffusion to permit the asymmetric auxin gradient essential for phototropic responses in *Arabidopsis thaliana*. At the same time, however, it should be noted that the auxin-dependent control of callose deposition and plasmodesmal gating is mediated by the auxin-regulated gene expression (including the auxin response factor 7, ARF7) and thus may be influenced by specific tissue-, age-, and environmental or experimental factors. Still however, the relationship between an enhanced expression of PIN2-LPs and increased levels of auxin (both associated with massive callose deposits and plasmodesmatal modifications in cell walls between asynchronous parts of antheridial filaments) suggests that a kind of functional integration among all these structural and regulatory elements might have appeared long before higher land plants had evolved. All the more so, biological significance of our observations in *C. vulgaris* remains to be clarified.

**Fig. 9:**
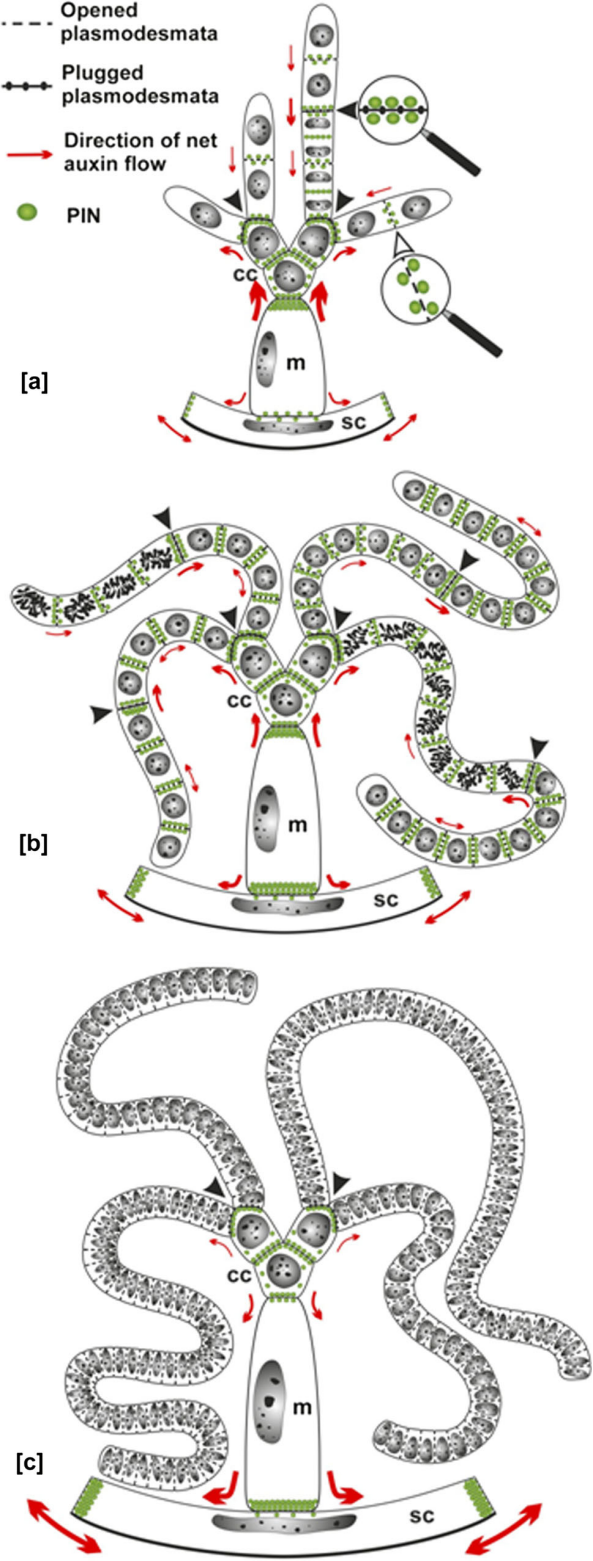
A schematic summary of the data obtained by immunofluorescence detection of PIN2-LPs in antheridial cells of *C. vulgaris*. [*a*] Early, [*b*] middle, and [*c*] late stages of spermatogenesis (including terminal differentiation of sperm cells); *cc* capitular cells, *m* manubrium, *sc* shield cell. Relative amounts of PIN2-LPs and their distribution modes (equal or polarized) are indicated by different numbers of round dots placed near each cell wall. Plugged (*black arrowheads*) and open plasmodesmata in cell walls adjoining, respectively, asynchronous and synchronous cells within individual antheridial filaments are distinguished by magnifying glasses. The thickness of *arrows* corresponds to the presumed dynamics of net PIN2-LPs-mediated auxin export

Different nuclear DNA contents were found to play a crucial role in designing structural and functional organization of non-generative component parts in the male reproductive organs of *Chara*: capitular cells, manubria, and shield cells (Maszewski 1991). Immunofluorescent localization of PIN2-LPs in the antheridia of *C. vulgaris* suggests that, by means of auxin-mediated pathways, additional layers of regulation have evolved to arrange supplementary control and integration mechanisms at the supra-cellular level. Despite extensive ultrastructural changes observed throughout successive periods of spermatogenesis (Kwiatkowska and Maszewski 1987a), capitular cells were found relatively most stable with respect to the high expression levels of PIN2-LPs, large amounts of IAA and abundant callose deposits (see also Godlewski 1988). Thus, functioning as reservoirs localized in the most central region of an antheridium, capitular cells might also be active (yet still presumably) in the synthesis and sequestration and/or distribution of auxin transported into and from one location to another. In contrast to capitular cells, cylindrical and elongated manubria reveal dynamic mobility of PIN2-LPs; first they localize centripetally (oriented mainly towards the capitular cells), then bipolarly, and in the end centrifugally, after changing their allocation towards the outer layer of the shield cells (Fig. 9). The ultimate positioning of PIN2-LPs in manubria may appear correlated with the lowered PIN2-LPs/IAA levels observed in antheridial filaments during terminal differentiation of the germ-line cells and with an increased IAA concentration in the shield cells prior to their opening and release of spermatozoids from ripe antheridia. Accordingly, the enhanced quantities of PIN2-LPs allocated at the margins of mature triangle-shaped shields cells might contribute to excessive accumulation of auxin, which is known to soften cell walls via modifications of pectins (Braybrook and Peaucelle 2013; Nafisi et al. 2015) and many of the physical properties of other cell wall components (Lewis et al. 2013).

The most important results of our observations are summarized in Fig. 9. Despite the highly developed antheridial cells can exchange molecular components and signaling factors directly via an elaborate system of plasmodesmatal connections (Kwiatkowska 1999) and the membrane trafficking pathways (including exocytosis and endocytosis; e.g., Hoepflinger et al. 2013), PIN2-LPs seem to provide additional regulatory support to create morphogenetic fields that influence spermatogenesis in *C. vulgaris*. During the early developmental stages of its male sex organs (in the proliferative period, when spermatids multiply by successive mitotic divisions), the net auxin flow is directed predominantly towards the central group of capitular cells and antheridial filaments (Fig. 9[a]). At later stages (Fig. 9[b, c]), relocalization of PIN2-LPs changes the directionality of auxins centrifugally towards shield cells. This, in turn, may eventually contribute to their opening and release of mature sperm cells. Biochemical mechanisms by which such a relocalization occurs (both in generative and non-generative antheridial cells of *Chara*) opens a new field of exploration.

### **Author contribution statement**

A.Ż. and J.M. equally participated in the conception and design of the study. A.Ż. contributed also to acquisition of the results and performed most of the analyses. P.P., J.J., M.N., and K.W. contributed to the qualitative and quantitative analysis of the microscopic data. J.P. is an author of the scheme and contributed to the revision of the manuscript. J.M. prepared the English version of the text. 

## Abbreviations

DiOC6(3): 3,3′-Dihexyloxacarbocyanine Iodide
ER: Endoplasmic reticulum
GSL8: GLUCAN SYNTHASE LIKE 8
IAA: Indole-3-acetic acid
IP: Immunoprecipitation
PAT: Polar auxin transport
PIN2: PIN-FORMED2
PIN2-LPs: PIN2-like proteins
